# Response to mTOR inhibition: activity of eIF4E predicts sensitivity in cell lines and acquired changes in eIF4E regulation in breast cancer

**DOI:** 10.1186/1476-4598-10-19

**Published:** 2011-02-14

**Authors:** Sampoorna Satheesha, Victoria J Cookson, Louise J Coleman, Nicola Ingram, Brijesh Madhok, Andrew M Hanby, Charlotte AB Suleman, Vicky S Sabine, E Jane Macaskill, John MS Bartlett, J Michael Dixon, Jim N McElwaine, Thomas A Hughes

**Affiliations:** 1 Leeds Institute of Molecular Medicine, St. James's University Hospital, Leeds University, Leeds, LS9 7TF, UK; 2 Department of Histopathology, St. James's University Hospital, Leeds, LS9 7TF, UK; 3 University of Edinburgh Cancer Research Centre, Institute of Genetics & Molecular Medicine, Edinburgh, EH4 2XR, UK; 4 Edinburgh Breakthrough Research Unit, Institute of Genetics & Molecular Medicine, University of Edinburgh, Edinburgh, EH4 2XR, UK; 5 Department of Applied Mathematics and Theoretical Physics, Cambridge University, Cambridge, CB2 0WA, UK

## Abstract

**Background:**

Inhibitors of the kinase mTOR, such as rapamycin and everolimus, have been used as cancer therapeutics with limited success since some tumours are resistant. Efforts to establish predictive markers to allow selection of patients with tumours likely to respond have centred on determining phosphorylation states of mTOR or its targets 4E-BP1 and S6K in cancer cells. In an alternative approach we estimated eIF4E activity, a key effector of mTOR function, and tested the hypothesis that eIF4E activity predicts sensitivity to mTOR inhibition in cell lines and in breast tumours.

**Results:**

We found a greater than three fold difference in sensitivity of representative colon, lung and breast cell lines to rapamycin. Using an assay to quantify influences of eIF4E on the translational efficiency specified by structured 5'UTRs, we showed that this estimate of eIF4E activity was a significant predictor of rapamycin sensitivity, with higher eIF4E activities indicative of enhanced sensitivity. Surprisingly, non-transformed cell lines were not less sensitive to rapamycin and did not have lower eIF4E activities than cancer lines, suggesting the mTOR/4E-BP1/eIF4E axis is deregulated in these non-transformed cells. In the context of clinical breast cancers, we estimated eIF4E activity by analysing expression of eIF4E and its functional regulators within tumour cells and combining these scores to reflect inhibitory and activating influences on eIF4E. Estimates of eIF4E activity in cancer biopsies taken at diagnosis did not predict sensitivity to 11-14 days of pre-operative everolimus treatment, as assessed by change in tumour cell proliferation from diagnosis to surgical excision. However, higher pre-treatment eIF4E activity was significantly associated with dramatic post-treatment changes in expression of eIF4E and 4E-binding proteins, suggesting that eIF4E is further deregulated in these tumours in response to mTOR inhibition.

**Conclusions:**

Estimates of eIF4E activity predict sensitivity to mTOR inhibition in cell lines but breast tumours with high estimated eIF4E activity gain changes in eIF4E regulation in order to enhance resistance.

## Background

Rapamycin is an immunosuppressant drug prescribed for prophylaxis of organ rejection following renal transplant [1]. Recently it, and derivatives such as everolimus, have been tested as cancer therapeutics with some success [2-5]. The drugs inhibit the serine/threonine-specific protein kinase mTOR (mammalian Target Of Rapamycin) by forming a complex with another protein, FKBP12 (FK 506-binding protein of 12 kDa), that then associates with mTOR. This association allosterically inhibits mTOR's ability to assemble the functionally active complex mTORC1 (mTOR complex 1) [6,7]. In addition, at high doses the drugs can bind directly to mTOR inhibiting its function [8]. mTORC1 activity is up-regulated in many cancers as a result of loss of function of tumour suppressor genes such as p53 or LKB1, up-regulation of AKT, or mitogenic signalling [9-11]. Pathways downstream of mTORC1 that contribute to carcinogenesis have also been defined. The main mTORC1 targets are the eIF4E-binding proteins (4E-BP1, 2 and 3) and the S6 protein kinases (S6K1 and 2) [12,13]. Hypophosphorylated 4E-BPs bind to and inhibit the translation factor eIF4E, while these interactions are inhibited by mTORC1-dependent 4E-BP phosphorylation, releasing active eIF4E [14]. S6K activity is stimulated by phosphorylation by mTORC1. The result of increased activity of both eIF4E and S6K is changes in translation. Increased eIF4E activity enhances cap-dependent translation of mRNAs with a high degree of secondary structure within their 5' untranslated regions (UTRs) [15,16], a subset of transcripts greatly enriched for cancer-related messages [17]. In addition, nuclear export of some cancer-related transcripts is stimulated by highly active eIF4E [18,19]. Increased S6K activity leads to up-regulation of overall translational capacity, as a result of increased ribosome biogenesis, and may also contribute to enhanced translation of transcripts with structured 5'UTRs via up-regulation of the activity of the translation factor eIF4A [20]. Therefore, increased mTORC1 activity in cancer enhances expression of key oncogenes and increases cellular growth potential. Reversing these effects, and thereby reducing cell growth or inducing apoptosis, is thought to be the basis of the therapeutic action of mTOR inhibitors in cancer.

However, mTOR inhibitors have proved less successful in cancer clinical trials than might be hoped from the importance of the molecular pathways involved [2]. This relates partly to some toxicity in non-target tissues [21,22], but also to intrinsic or acquired resistance in many individual cancers. Consequently, there is a need for predictive biomarkers to allow selection of patients with cancers most likely to respond to such agents. A number of potential biomarkers have been discussed in the literature, focusing on expression levels or phosphorylation states of mTOR itself [23], or the immediate targets of mTORC1, 4E-BP1 [24,25] and S6K1 [26,27]. Here, we take a different approach and estimate the *activity *of eIF4E, one of the key effectors of mTORC1 function, and investigate whether this reflects response to mTOR inhibition in both tissue culture and in clinical breast cancers.

## Methods

### Cell culture, transfection, proliferation assays

Cell lines were obtained from American Tissue Culture Collection or European Collection of Animal Cell Cultures and were maintained at 37°C in humidified air/5% CO_2_. Bi-monthly mycoplasma checks (MycoAlert assay; Lonza, Basel, Switzerland) were consistently negative. Cell-specific culture/transfection conditions are described in Additional file 1, Table S1. Plasmids pTH-GFPa (control GFP reporter) [28], GFP+60 (structured GFP reporter) [29] and pcDNA3HA-eIF4E [30] have been described previously. For proliferation assays, cells were plated into 96-well, flat-bottomed plates at 5 × 10^3^-2 × 10^4 ^cells/well (depending on cell line growth characteristic to ensure continued log phase growth; this was assessed by examination of growth of DMSO-treated cells over 48 h). Five replicate wells were treated with DMSO (control) or InSolution™ Rapamycin (Calbiochem, Darmstadt, Germany) for 24 (data not shown) or 48 h. Metabolically active cells were quantified by assessing conversion of 3-(4,5-Dimethyl-2-thiazolyl)-2,5-diphenyl-2H-tetrazolium bromide (Sigma-Aldrich, Dorset, UK) to formazan. Formazan was dissolved in propan-1-ol and quantified as absorbance at 570 nm (Opsys, Dynex, Sussex, UK).

### Western blotting

Proteins were extracted in RIPA (50 mM Tris HCl pH 7.4, 150 mM NaCl, NP40 1%, Complete inhibitors [Roche, Basel, Switzerland]) and were quantified in triplicate with the RCDC protein assay (BioRad, Hercules, USA). 20 μg of protein was loaded into wells of 12% NuPAGE bis-tris gels running in MOPS NuPAGE buffer (Invitrogen, Paisley, UK). Proteins were transferred to PDVF membrane (Millipore, Billerica, USA) in NuPAGE transfer buffer (Invitrogen, Paisley, UK). Membranes were blocked and incubated with antibodies in 5% dried milk in TBS-T (Tris-buffered saline-0.1% Tween 20) and were washed in TBS-T. Primary antibodies: rabbit monoclonal anti-phosphoThr37/46 4E-BP1, 1:500, and rabbit polyclonal anti-4E-BP1, 1:500 (#2855 and #9452, Cell Signalling Technology, Beverly, USA); mouse monoclonal anti-eIF4E, 1:500 (sc9976, Santa Cruz, USA). We have previously validated specificities of these antibodies, including the phospho-specificity of the anti-phospho clone [31], although we cannot exclude that the anti-phospho-4E-BP1 may cross-react with phospho-4E-BP2 or 3. Secondary antibodies: anti-mouse/rabbit HRP conjugates, 1:1000 (DAKO, Glostrup, Denmark). Proteins were detected using Supersignal West Femto (Thermo Fisher, Waltham, USA) and Chemidoc XRS (BioRad, Hercules, USA), and analysed using ImageJ 1.42q (NIH, http://rsb.info.nih.gov/ij).

### Translational efficiency assay

We have previously reported the protocol in considerable detail [32,33]. In brief, relative GFP protein and mRNA levels were used to calculate amounts of GFP protein produced per unit mRNA. RNA was purified using RNeasy (Qiagen, Crawley, UK) and contaminating DNA was removed with TURBO DNase I (Applied Biosystems, Warrington, UK). cDNA was synthesized from oligo-dT primed RNA using Superscript II (Invitrogen, Paisley, UK). Real-time PCR reactions were performed in triplicate using SYBR Green PCR Master Mix on an ABI7900HT machine (Applied Biosystems, Warrington, UK). GFP mRNA levels were normalized to those of the reference gene RPLP0 [34] and relative expression calculated using the ΔΔCt method [35]. For analysis of GFP protein expression, cells were suspended in media containing 1% serum and fluorescence quantified (mean fluorescent intensity of 10^4 ^events after exclusion of debris/dead cells on forward activated light scatter/side scatter) at 525 nm using an LSRII machine (BD Biosciences, Oxford, UK).

### Ethical issues, patient material, immunohistochemistry

Ethical permissions were obtained from Northern and Yorkshire MREC (4/MRE03/89) and Leeds East REC (05/Q1206/136). Postmenopausal female patients with operable early breast cancer (T1-3, N0-1, M0) proceeding to primary surgery were recruited, written informed consent was taken, and patients were treated as previously described in detail [36]. In brief, core biopsies were taken at time of presentation with a palpable breast lump and were processed for diagnostic assessments. Patients were given 11-14 days of everolimus 5 mg once daily immediately before tumour resection. Excision specimens were processed by the pathology laboratory for diagnostic tests. Clinical/pathological details of patients are listed elsewhere [36]. Matched biopsy and excisional tumour blocks from 22 patients were used. Immunohistochemistry was performed for Ki67 (mouse monoclonal clone MIB-1; Dako, Glostrup, Denmark), 4E-BP2 (rabbit polyclonal #2845, Cell Signalling Technology, Beverly, USA), eIF4E, 4E-BP1 and phosphoThr37/46 4E-BP1 (as for Westerns) exactly as described and validated previously [31,36] on single sections from each case for each antigen. Ki67 was quantified using a previously validated protocol for scoring percentages of stained cells as proportions of total cancer cells [37], and these data have been published [36]. Other markers were scored by two independent individuals (VJC, CS) taking into account average intensity and percentage of positively stained tumour cells. Intensity scores (0 no staining, 1 weak, 2 moderate and 3 strong) were added to percentages scores (1 <5%, 2 6-25%, 3 26-75% and 4 >75%) giving totals of 0 or 2-7. Consensus scores were determined for sections with different initial scores; scoring was overseen by a consultant breast histopathologist (AMH).

### Statistics

Analyses were performed using Student's T Test, Spearman's rho correlation, or linear regression in Excel v12 (Microsoft, Redmond, USA), SPSS v15 (SPSS, Chicago, USA) and MATLAB (MathWorks, Natick, USA). Tests were two sided; p < 0.05 was considered to indicate significance.

## Results

### Cell lines show a range of sensitivities to rapamycin

**Figure 1:**
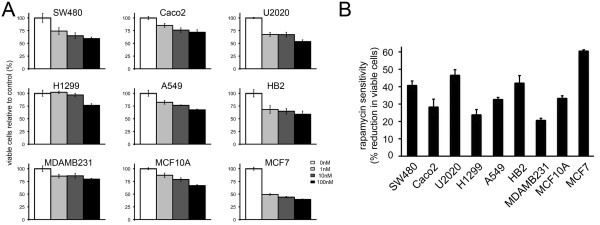
**Sensitivities of cell lines to rapamycin vary by up to three fold**. A) Cells were treated with either control, or different doses of rapamycin and growth/proliferation was monitored using MTT assays. MTT readings after 48 hours are shown relative to control. B) Relative sensitivities to rapamycin are shown; these are the % reductions in growth/proliferation caused by 100 nM rapamycin. Data points represent means (+/- standard deviations) from five independent wells of cells within a representative experiment. Data from independent repeat experiments are presented in Additional file 2, Figure S1 to demonstrate the reproducibility of these measurements in each cell line.

Rapamycin and its derivatives induce a broad range of responses when used as cancer therapeutics with growth of some cancers reduced while others are resistant. We were interested to examine this variation, therefore we treated a panel of cell lines with rapamycin and determined drug sensitivities. The panel was representative of the three most common cancers in the UK: colorectal (SW480 and Caco2, moderately-differentiated and heterogeneous colon cancer lines respectively); lung (U2020, small cell, and H1299 and A549, non small cell cancer lines); and breast (MCF7 and MDA-MB-231, luminal and basal breast cancer lines respectively). In addition, two immortal breast epithelial lines of non-cancer origin (HB2 and MCF10A) were examined in order to allow study of potential differential sensitivity between cancer and non-cancer cells. We treated cells with doses of rapamycin and determined proliferation/survival relative to control treated cells using MTT assays after 48 h (Figure 1A). Sensitivities to the highest dose are shown in Figure 1B. As expected a range of sensitivities were seen, with a three fold difference between the most sensitive (MCF7) and most resistant (MDA-MB-231). Cells of non-cancer origin (HB2 and MCF10A) were found to have sensitivities between these extremes.

### The phosphorylation state of 4E-BP1 does not predict rapamycin sensitivity

**Figure 2:**
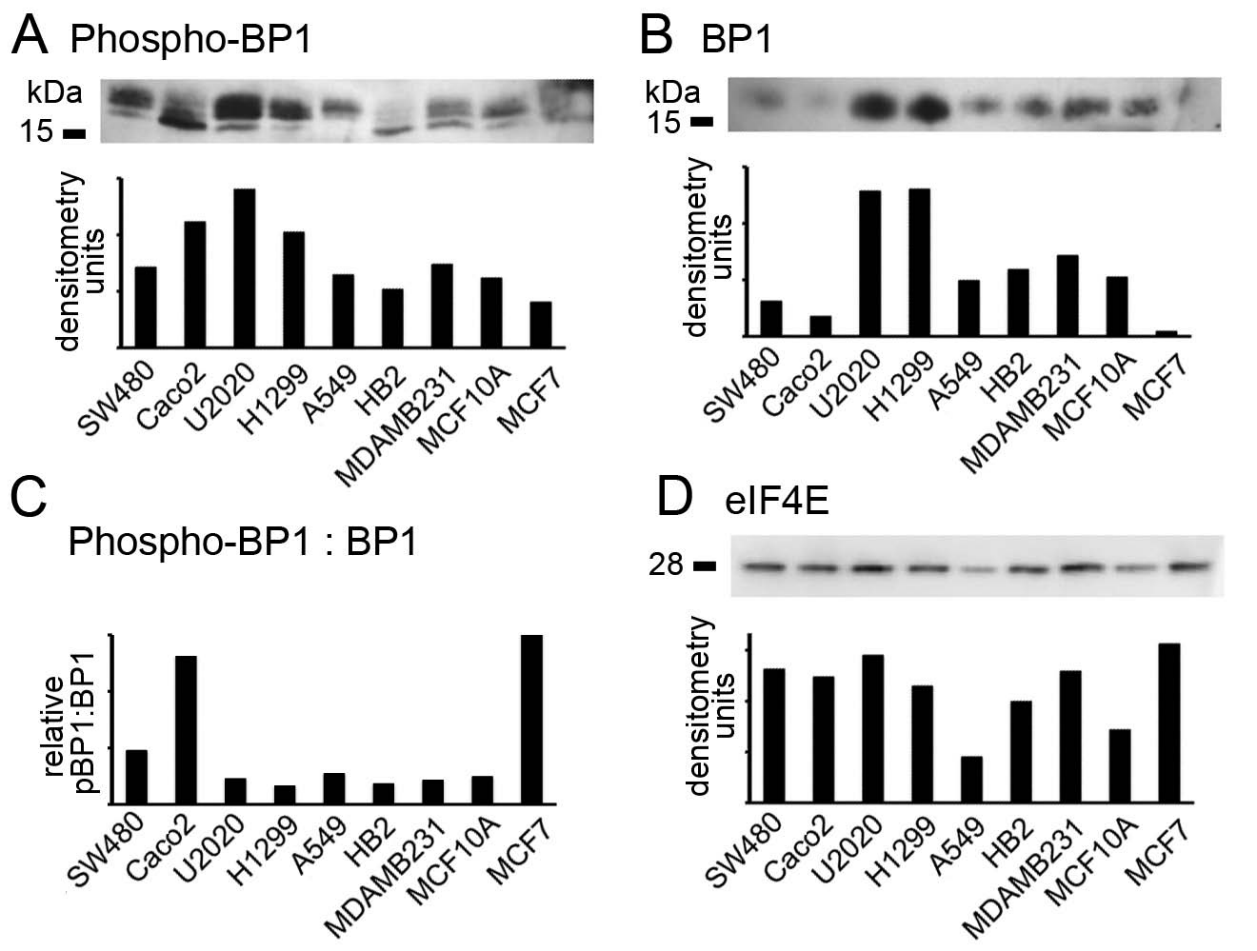
**Levels of 4E-BP1 phosphorylation do not predict rapamycin sensitivity**. Expression of phospho-4E-BP1 (A), total 4E-BP1 (B) and eIF4E (D) was examined in the cell lines shown by Western blot. Relative ratios of phospho-4E-BP1 to total 4E-BP1 are shown in C. Equal masses of total protein were loaded in each lane as determined by triplicate measurements with colourimetric protein mass assays, and expression was quantified by densitometry.

Next, we aimed to identify molecular markers that correlated with these sensitivities, therefore that might represent predictive biomarkers for mTOR inhibitors. Potential biomarkers have previously been proposed; of particular interest was the phosphorylation status of 4E-BP1 [24] since the 4E-BP1/eIF4E axis has been shown to be critical for mTOR-mediated transformation [38]. 4E-BP1 is directly phosphorylated by mTORC1 [12], potentially leading to increased eIF4E activity and enhanced translation of cancer-related transcripts [15-17]. Thus, levels of phosphorylated 4E-BP1 may reflect contributions of mTORC1-signalling to cancer-associated translational deregulation, and consequently the sensitivity of such deregulated cells to mTOR inhibition. We performed Western blot analysis of levels of mTORC1-dependent 4E-BP1 phosphorylation (Thr37/Thr46) in the same cell lines as before (Figure 2A). At least three different phosphorylated 4E-BP1 (phospho-4E-BP1) species were seen, representing various combinations of the seven potential phosphorylation events [39-41]. We found no correlation between phospho-4E-BP1 and rapamycin sensitivity (compare Figures 2A and 1B). However, levels of phospho-4E-BP1 reflect not only mTORC1 activity but also levels of overall 4E-BP1, therefore we also analysed total 4E-BP1 expression (Figure 2B) and determined the ratios of phospho- to total 4E-BP1 (Figure 2C) as a measure of mTORC1's influence on 4E-BP1 function, as previously reported [25]. We found no correlation between this measure and rapamycin sensitivity. Finally, 4E-BP1's influence on cellular behaviour is determined by the amount of eIF4E remaining unbound by 4E-BP1, consequently, variation in eIF4E expression would also have a critical role. We analysed eIF4E expression and found greater than three fold variation in eIF4E expression (Figure 2D). We concluded that levels of phospho-4E-BP1 do not correlate with functional influences of mTORC1 on cap-dependent translation, partly as substantial variations in total 4E-BP1 and eIF4E expression mask this direct relationship. A measure of eIF4E activity would, however, take into account the variation in expression of all these components and might provide a better predictive marker.

### Assessment of translational efficiencies defined by a structured 5'UTR; estimation of eIF4E activity

**Figure 3:**
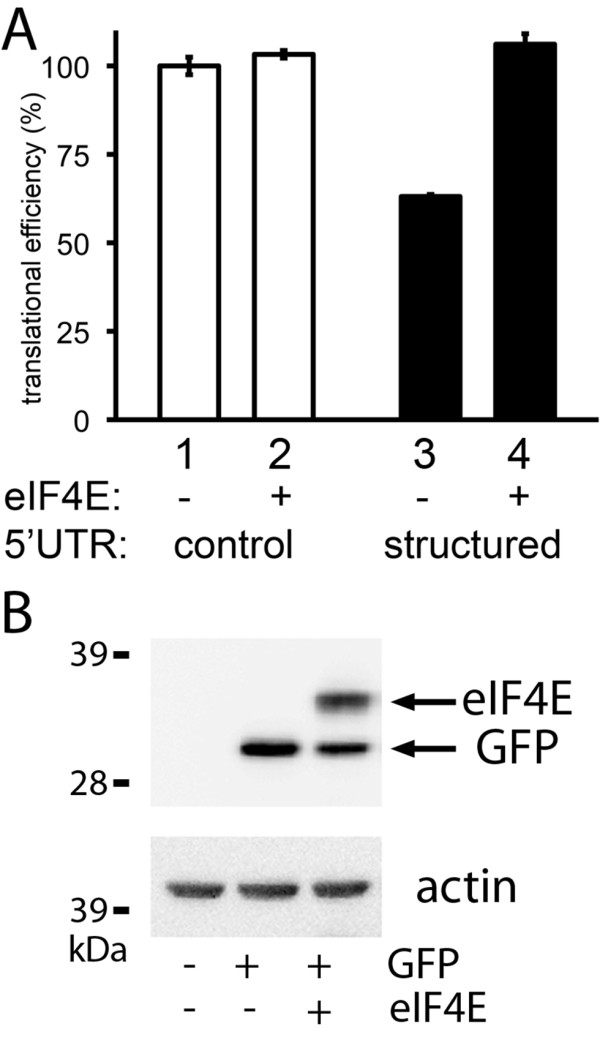
**Translational efficiency specified by a structured 5'UTR reporter responds to changes in eIF4E activity**. A) Reporters were constructed to express mRNAs containing the GFP reading frame preceded by a control 5'UTR lacking regulatory motifs (control) or a sequence predicted to form a stable stem-loop structure (structured). MCF7 cells were transiently transfected with equal copy numbers of either control or structured reporters along with either empty expression vector (-) or vector to allow over-expression of eIF4E (+). GFP protein and mRNA were quantified by flow-cytometry and real-time PCR respectively. Translational efficiency (protein synthesised per unit mRNA) is presented relative to the control. Data points represent means (+/-standard deviations) of values from three separate wells of cells within a representative experiment. B) Expression of exogenous proteins was confirmed within cells transfected with control GFP reporter and vector to allow over-expression of eIF4E as shown by Western blot analysis for the HA-epitope tag; exogenous GFP and eIF4E both include this epitope. Note that the small reduction in GFP protein associated with eIF4E-transfection does not result from a change in translational efficiency (see Panel A).

Our hypothesis was that assessment of the *activity *of a key mTORC1 regulated pathway gives direct insights into the contribution of deregulated mTORC1 to cellular behaviour and therefore, potentially, into likely sensitivity to mTOR-inhibitors. A critical effect of up-regulated mTORC1 is to up-regulate eIF4E activity thereby enhancing translational efficiencies of transcripts with structured 5'UTRs [15]; therefore, we designed an assay to measure these translational efficiencies. We have previously shown that a 5'UTR from human axin2 transcripts contains a sixty nucleotide sequence that is predicted to form a stable stem-loop structure [29]. This sequences meets the criteria associated with UTRs that determine differential translational efficiencies in response to changes in eIF4E activity [16], while lacking other translation regulatory motifs (e.g. upstream AUG codons or binding sites for trans-acting proteins). In addition, we previously demonstrated that this sequence determined cell type specific translational efficiencies [29]. We now wished to examine whether the translational efficiency defined by this sequence would respond to changes in eIF4E activity, and could therefore be representative of mTORC1's influence on cap-dependent translation of structured transcripts. The sequence was cloned upstream of the GFP reading frame in an expression vector. MCF7 cells were transiently transfected with an equal copy number of vectors to allow expression of GFP mRNAs with either a control non-regulatory 5'UTR or this sequence as a 5'UTR, along with either empty expression plasmids or plasmids allowing eIF4E over-expression. GFP protein expression was measured by flow-cytometry and GFP mRNA expression was measured by qPCR allowing determination of relative translational efficiencies for each GFP message as previously described [32,33] (Figure 3A). Western blot analyses were used to confirm expression of exogenous eIF4E and GFP (Figure 3B). The translational efficiency of the control reporter was not significantly altered by eIF4E over-expression (compare lanes 1 and 2), demonstrating that eIF4E over-expression did not cause a general enhancement of translation. As previously reported [29], the structured 5'UTR conferred repression of translation (compare lanes 1 and 3; p = 0.002). Critically, this repression was overcome by exogenous eIF4E (compare lanes 3 and 4; p = 0.002), resulting in translation with the same efficiency as messages lacking inhibitory 5'UTRs. We concluded that this reporter did indeed respond to changes in eIF4E activity and thus can be used to provide an estimate of eIF4E-dependent translation from structured 5'UTRs.

### Estimates of eIF4E activity predict rapamycin sensitivity in tissue culture cells

**Figure 4:**
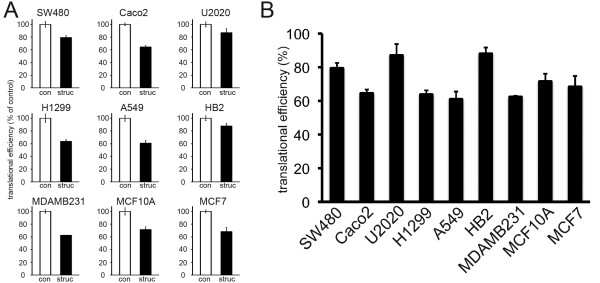
**eIF4E activities vary in different cell lines**. A) Cells were transiently transfected with equal copy numbers of plasmids to allow expression of transcripts with the GFP reading frame preceded by either a control 5'UTR lacking regulatory motifs (con) or the eIF4E-responsive structured 5'UTR (struc). GFP protein and mRNA were quantified by flow-cytometry and real-time PCR respectively. Translational efficiency (protein synthesised per unit mRNA) is presented relative to the control. Data points represent means (+/-standard deviations) of values from three separate wells of cells from two independent experiments (a total of six values). B) Translational efficiencies of transcripts with eIF4E-responsive 5'UTRs in the 9 cells lines.

**Figure 5:**
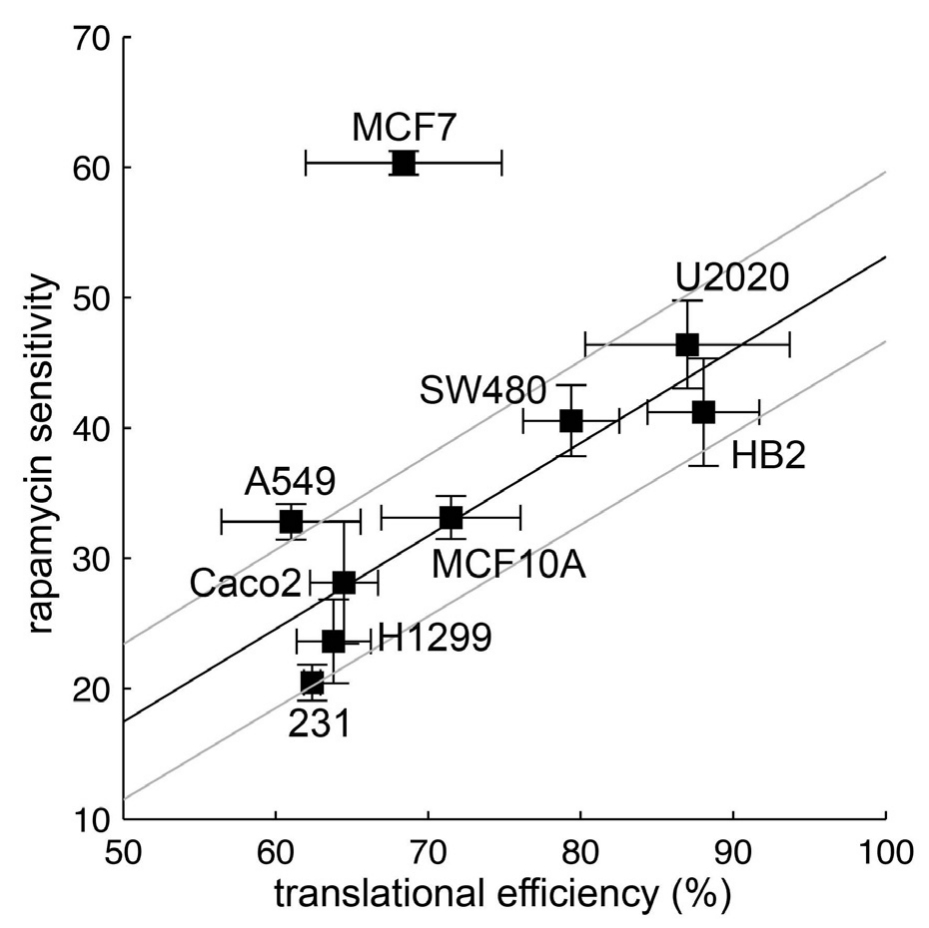
**Experimentally determined eIF4E activities correlate with sensitivities to rapamycin**. Data from Figures 4B and 1B were plotted for each cell line as labelled. Linear regression was performed to determine the relationship in the cell lines excluding the outlier, MCF7 cells; the linear model is shown as a line (black) with 95% confidence intervals (grey lines) (p = 0.0037).

Relative translational efficiencies specified by this eIF4E-responsive 5'UTR were determined in the panel of cell lines. Cells were transiently transfected with vectors to allow expression of GFP mRNAs with control or the structured 5'UTR as before, and translational efficiencies were determined (Figure 4A). A range of translational efficiencies was seen, with A549 cells determining the lowest, and HB2 cells the highest (Figure 4B). Surprisingly, the two cells lines of non-cancer origin (HB2 and MCF10A) were found to determine relatively efficient translation from the reporter 5'UTR. Importantly, translational efficiencies specified by the eIF4E-responsive 5'UTR correlated with rapamycin sensitivity (Figure 5). Initially, we analysed this relationship using Spearman's rank correlation coefficient; we found a strong and significant positive association (r = 0.72; p = 0.037). However, the correlation was particularly evident in 8/9 cell lines; if MCF7 cells, which were more sensitive to rapamycin than predicted, were excluded the strength and significance of the relationship was increased (r = 0.83; p = 0.015). Similarly in linear regression, a highly significant relationship was seen when MCF7 cells were excluded from the analysis (Figure 5; p = 0.0037).

### Estimated eIF4E activity in breast tumours does not predict reduced tumour proliferation after preoperative treatment with the rapamycin derivative everolimus

**Figure 6:**
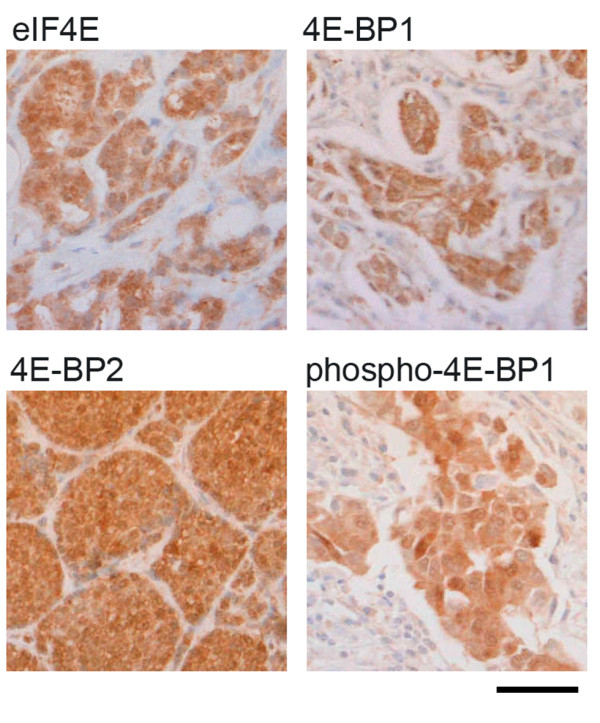
**Representative tumour sections showing immunoreactivity as labelled**. Staining within these sections was scored as eIF4E 6, 4E-BP1 6, 4E-BP2 6 and phospho-4E-BP1 5. Scale bar represents 0.05 mm.

Next, we wished to examine whether eIF4E activities within tumour cells predict clinical responses to mTOR inhibition in cancer patients, and whether changes in eIF4E activities after treatment reflect these responses. Everolimus is a rapamycin-derivative with improved oral bioavailability that is currently undergoing trials as a cancer therapeutic. We recently performed a clinical trial examining effects of everolimus on tumour cell proliferation when given preoperatively as a single agent to breast cancer patients [36]. By examining Ki67 staining in matched pre-treatment biopsy and post-treatment excisional samples, we found that 5 mg everolimus daily for up to 14 days was significantly associated with reduced tumour cell proliferation. Unfortunately, it is not possible to estimate eIF4E activities directly in tumour samples such as these using the reporters described above. As an alternative we estimated eIF4E activity from expression and phosphorylation states of multiple regulators of the translational initiation pathway (Figure 6; Table 1). We have previously described the development and prognostic value of this estimate in breast tumours [31]. Expression levels of eIF4E, 4E-BP1, 4E-BP2 and Thr37/Thr46 phosphorylated 4E-BP1 (phospho-4E-BP1) within tumour cells were determined semi-quantitatively in matched pre- and post-treatment tumour samples from 22 patients using immunohistochemistry. Activity of eIF4E was estimated by combining these scores to reflect the inhibitory influence of 4E-BPs on eIF4E, and the activating influence of 4E-BP1 phosphorylation on eIF4E, using a formula previously derived from regression modelling of individual contributions of each component to prognosis in a large cohort of breast cancer patients: X-B1/4+PB1/2-B2/4, where X, B1, PB1 and B2 represent scores for eIF4E, 4E-BP1 phospho-4E-BP1 and 4E-BP2 respectively [31]. We previously showed that this measure gave improved insights into the prognostic influence of eIF4E by reflecting eIF4E activity more accurately than examining eIF4E expression levels alone.

**Table 1:** Expression scores for eIF4E (4E), 4E-BP1 (BP1), 4E-BP2 (BP2), phospho-4E-BP1 (pBP1) and Ki67, and estimates of eIF4E activity in matched pre- and post-treatment samples from patients treated with 11-14 days of everolimus.

	pre-treatment						post-treatment					
**Patient**	**4E**	**BP1**	**BP2**	**pBP1**	**activity**	**Ki67**	**4E**	**BP1**	**BP2**	**pBP1**	**activity**	**Ki67**

1	6	5	5	6	6.5	15.5	0	0	6	0	-1.5	23.1
2	3	5	5	5	3	6.0	0	3	5	0	-2	0.9
3	3	3	5	4	3	7.6	5	4	5	3	4.25	2.7
4	5	6	6	6	5	22.1	6	7	7	6	5.5	16.7
5	4	6	4	6	4.5	44.6	4	4	4	3	3.5	29.7
6	4	0	6	6	5.5	34.2	5	5	5	4	4.5	23.0
7	3	6	5	5	1.75	4.2	4	4	4	0	2	2.7
8	4	3	4	2	3.25	1.6	3	0	4	3	3.5	4.4
9	5	7	7	6	4.5	15.3	5	6	7	5	4.25	6.8
10	5	7	6	5	4.25	22.6	5	3	6	2	3.75	6.5
11	5	0	6	2	4.5	18.4	5	0	4	0	4	7.4
12	5	3	6	6	5.75	3.6	6	6	7	3	4.25	47.2
13	4	4	0	5	5.5	10.1	6	5	5	2	4.5	3.6
14	6	4	0	6	8	17.4	4	4	6	2	2.5	10.1
15	5	0	0	6	8	10.2	4	0	7	5	4.75	17.8
16	5	4	0	4	6	10.6	6	4	6	2	4.5	13.0
17	4	4	2	6	5.5	16.2	6	3	5	4	6	8.4
18	0	0	0	4	2	6.2	4	2	7	0	1.75	5.2
19	4	4	0	5	5.5	18.2	7	7	5	3	5.5	9.0
20	6	4	0	7	8.5	18.0	5	6	7	5	4.25	3.8
21	5	4	6	6	5.5	20.0	0	0	5	5	1.25	13.4
22	5	4	2	4	5.5	6.8	4	4	7	3	2.75	5.3

17/22 tumours showed reduced Ki67 scores after treatment (mean reduction 48%) indicating apparent responses to everolimus (Table 1). Disappointingly, estimates of pre-treatment eIF4E activity did not predict the occurrence or extent of these responses. Similarly, pre-treatment phospho-4E-BP1 levels had no predictive value. Estimated eIF4E activity was, however, reduced in post-treatment samples (mean change in score -1.7; range -8 to +1.25; p < 0.001) but surprisingly this was not attributable to reduced phospho-4E-BP1. Phospho-4E-BP1 expression was reduced after treatment (mean -2.3; range -6 to +1; p < 0.001), suggesting a reduction in mTOR-dependent phosphorylation of 4E-BP1, but this reduction in phospho-4E-BP1 was not significantly correlated with the reduction in estimated eIF4E activity, and changes in levels of the other components had strong influences on estimated eIF4E activity. For example, 4E-BP1 expression changed considerably (mean -0.3; range -5 to +5; p = 0.01), meaning that 8 individual decreases in phospho-4E-BP1 could be explained at least partially by reductions in total 4E-BP1, as opposed to reduced phosphorylation. This explanation is supported by observations that the phospho-4E-BP1 species examined here (Thr37/46) can be relatively resistant to mTOR inhibition [39]. 4E-BP2 expression also frequently changed (mean +2.2; range -2 to +7; p < 0.001), while some individuals showed dramatic changes in expression of eIF4E (mean -0.1; range -6 to +4). Interestingly changes in eIF4E and 4E-BP1 were positively associated (r = 0.60, p = 0.003), often resulting in relatively small changes in estimated eIF4E activity, despite substantial fluctuations in expression of the individual proteins. Critically, neither reduced estimated eIF4E activity or reduced phospho-4E-BP1 correlated with reduced Ki67.

### High estimated eIF4E activities in breast tumours are associated with everolimus-induced changes in eIF4E regulation

A striking observation was that post-treatment levels of eIF4E and the 4E-BPs frequently varied considerably from pre-treatment levels. Our hypothesis was that these changes represent development of acquired resistance to inhibition of eIF4E activity, by induction of changes in eIF4E regulation within tumour cells or by clonal selection of cells with different eIF4E-related expression profiles. Importantly, this proposed acquired resistance is not necessarily reflected in higher proliferation rates. Using this hypothesis, one would predict that tumours with high pre-treatment estimated eIF4E activities would be most subject to drug-induced expression changes or to clonal selection pressures, and would show the greatest adaptive changes in eIF4E regulation. In fact, high estimated eIF4E activity pre-treatment did positively correlate with the combined magnitude of changes in expression of the four markers (r = 0.46, p = 0.03) and, in particular, with increases in 4E-BP2 expression (r = 0.55, p < 0.01). We concluded that high estimated eIF4E activity may, in fact, predict tumour response to everolimus; however this response is not the expected reduction in proliferation, but is development of changes in eIF4E regulation, presumably to promote everolimus resistance.

## Discussion

The mTOR pathway, which promotes cell proliferation, presents an attractive target for cancer therapy since it is deregulated in a wide range of cancer types and a large proportion of cases of each type [2]. However, resistance of some cancers to mTOR-directed therapeutics has limited the success of mTOR inhibitors. We have examined this variation in response, initially, in cell lines. As expected, and in accordance with other published work [42-44], we found a range of sensitivities to rapamycin (Figure 1). Surprisingly, we found that cancer cell lines were not more sensitive than cells of non-cancer origin, despite the well established preferential sensitivity of cancer cells over non-cancer cells in animal models and, to an extent, in humans [3-5]. This observation most likely demonstrates that up-regulation of mTORC1, and consequently sensitivity to its inhibition, is actually associated with growth as opposed to malignancy, and therefore that highly-proliferative, immortal, non-cancer cell lines are un-representative of 'normal', relatively slowly growing, epithelial cells with respect to mTORC1 signalling. The efficacy of rapamycin as an immunosuppressant drug [1] and the side-effects seen in cancer therapies [21,22] support the view that proliferating cells are targeted.

Identification of predictive biomarkers for mTOR-targeted therapies such as rapamycin or everolimus has become a research focus [2]. Levels of phosphorylated mTOR, S6K1 or 4E-BP1 have been seen as logical markers as these phosphorylation events induce mTOR activity or are directly catalysed by mTORC1, and therefore levels may reflect the extent of mTORC1 deregulation. However, in principle, it is obvious that levels of these species may not correlate directly with their influences on down-stream signalling and consequent changes in cellular behaviour, since these influences would also be defined by expression/activity of the other regulatory molecules of the pathways. Despite this, some predictive value has been demonstrated for each marker [23,24,27]. We found levels of phosphorylated 4E-BP1, and the proportions of phospho-4E-BP1 within the total pool of 4E-BP1 to be unrelated to rapamycin sensitivity in tissue culture, in accordance with previous work in a cell line panel also containing MCF7 and MDA-MB-231 cells [42]. Moreover, we showed that this, in fact, was the expected result in the context of variation in 4E-BP1 and eIF4E expression (Figure 2). As an alternative predictive marker, we developed assays to estimate one of the key functional end-points of mTORC1 signalling, eIF4E *activity*. We found this estimate to be significantly associated with rapamycin sensitivity in cell culture. It was notable, however, that estimated eIF4E activity was the most significant predictor of rapamycin sensitivity for 8 of the cell lines (Figure 5), while MCF7 cells were twice as sensitive as predicted by this relationship. MCF7 cells over-express S6K1, on account of amplification of its gene [45]; one explanation for enhanced sensitivity in MCF7 cells may be that with constitutively high S6K1 activity, the cells are dependent upon mTOR-induced S6K functions such as more general translational effects [46]. In support of this, S6K1 over-expression has previously been associated with increased rapamycin sensitivity [42].

Importantly, we also examined whether estimates of pre-treatment eIF4E activity in clinical breast tumours predicted response to the mTOR inhibitor everolimus. Disappointingly and in contrast to our *in vitro *work, we found estimated eIF4E activity did not predict response to mTOR inhibition as assessed by change in tumour cell proliferation. However, we did find that pre-treatment eIF4E activity in tumours was significantly associated with substantial changes in the expression of eIF4E and its regulators post-treatment. We interpret this to suggest that cancers with high eIF4E activity may indeed have been sensitive to everolimus, as suggested by our *in vitro *data, but that the cells remaining after two weeks of drug treatment reflect selection to acquire drug resistance by changing the pathways regulating eIF4E function. Data show that this proposed resistance is not necessarily associated with lower estimated eIF4E activity or higher proliferative rates. This hypothesis highlights a difference between short-term (two day) sensitivity assays *in vitro *and longer term (two week) drug treatments in patients; in the latter case it is inevitably more difficult to assess the early response of tumour cells to treatment and there is considerable scope for acquired changes to take place. Finally, it is interesting to note that our data do not support the use of phospho-4E-BP1 as either a predictive or pharmacodynamic marker for mTOR inhibitors as some have attempted [2,21] since it is clear that changes in phospho-4E-BP1 relate not only to inhibition of 4E-BP1 phosphorylation, but also to dramatic changes in overall 4E-BP1 expression.

## Competing interests

JMD possesses an unrestricted educational grant and honoraria for seminar presentations from Novartis Pharma. The other authors declare that they have no conflicts of interest.

## Authors' contributions

SS, VJC, LJC and NI performed the *in vitro *experiments. BM and JNM performed statistical analyses. CABS, VJC and AMH performed semi-quantitative scoring of tissue sections. VSS, EJM, JMSB, JMD established the everolimus clinical trial, and obtained/analysed clinical samples. TAH devised and directed the study. All authors contributed to writing the manuscript and have approved the final version.

## Supplementary Material

Additional file 1 **Table S1**. Cell culture and transfection conditions. Click here for file

Additional file 2 **Figure S1**. Sensitivities of cell lines to rapamycin are reproducible. Relative sensitivities to 100 nM rapamycin are shown; these are the % reductions in growth/proliferation caused by the drug as compared to control treated cells. Data from Figure 1B are reproduced (filled bars) alongside independent repeat analyses (open bars). Data points represent means (+/- standard deviations) from five independent wells of cells. Click here for file
