# Supplementary material for: Response to mTOR inhibition: activity of eIF4E predicts sensitivity in cell lines and acquired changes in eIF4E regulation in breast cancer
Source: Mol Cancer. 2011 Feb 14;10:19. doi: 10.1186/1476-4598-10-19 (PMC3055230; doi:10.1186/1476-4598-10-19)
Supplement: Additional file 1 — Table S1. Cell culture and transfection conditions. [file 1476-4598-10-19-S1.DOC]

| **Cell Line** | **Culture conditions**  reagents: Invitrogen, Paisley, UK except FCS from Harlan Laboratories, Indianapolis, USA | **Transfection conditions**  1) Amount of reagent (Invitrogen, Paisley, UK)  2) Replacement medium 5h after addition of transfection mix |
| --- | --- | --- |
| A549 | D-MEM, 10% FCS  1mM glutamine | 1) 1.2μl/cm^2^ Optifect  2) standard medium with 20% FCS |
| Caco2 | D-MEM, 10% FCS  1mM glutamine  20mM HEPES  1% MEM non-essential amino acids | 1) 1μl/cm^2^ Lipofectamine 2000  2) standard media with 20% FCS |
| H1299 | RPMI 1640, 10% FCS  2.5mg/ml glucose  1mM sodium pyruvate  0.075% sodium bicarbonate  10mM HEPES | 1) 7.5µl/cm^2^ Lipofectamine 2000  2) standard medium with 20% FCS |
| HB2 | D-MEM, 10% FCS  1mM glutamine | 1) 0.75µl/cm^2^ Lipofectamine 2000  2) standard medium |
| MCF7 and MDAMB231 | RPMI 1640, 5% FCS | 1) 0.75µl/cm^2^ Lipofectamine 2000  2) standard medium |
| MCF10A | D-MEM/F-12 (1:1)  0.5μg/ml Hydrocortisone  0.1μg/ml Cholera Enterotoxin  5% Horse Serum | 1) 1.2μl/cm^2^ Optifect  2) standard medium with 10% Horse Serum |
| SW480 | RPMI 1640, 10% FCS  2.5mg/ml glucose  1mM sodium pyruvate  0.075% sodium bicarbonate  10mM HEPES | 1) 1μl/cm^2^ Lipofectamine 2000  2) standard media with 20% FCS |
| U2020 | RPMI 1640  10% FCS | 1) 0.75μl/cm^2^ Lipofectamine 2000  2) standard medium with 20% FCS |
