# Supplementary figures and images for: Response to mTOR inhibition: activity of eIF4E predicts sensitivity in cell lines and acquired changes in eIF4E regulation in breast cancer
Source: Mol Cancer. 2011 Feb 14;10:19. doi: 10.1186/1476-4598-10-19 (PMC3055230; doi:10.1186/1476-4598-10-19)

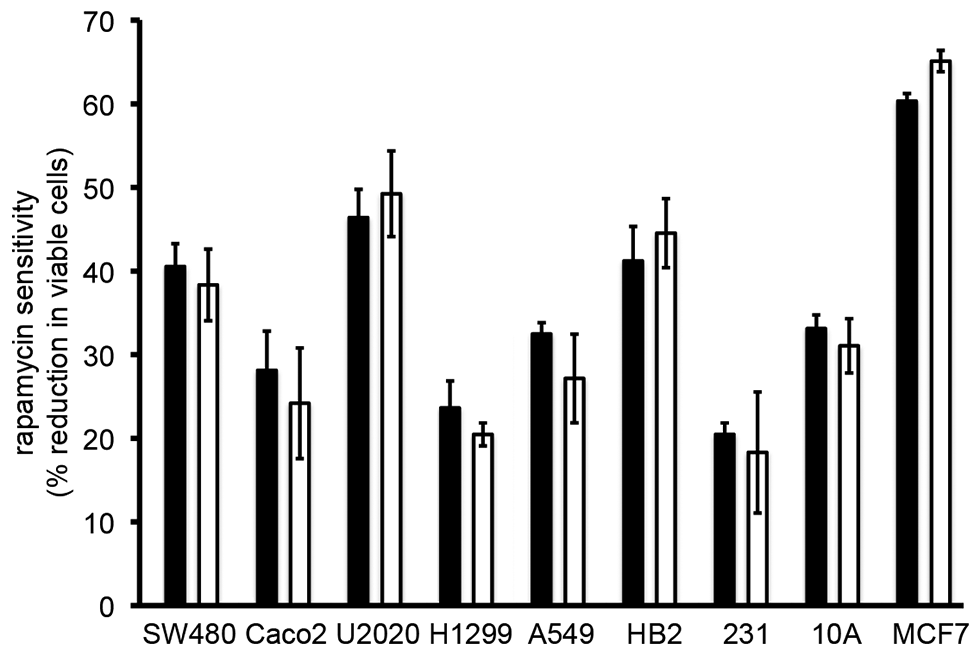

Supplement: Additional file 2 — Figure S1. Sensitivities of cell lines to rapamycin are reproducible. Relative sensitivities to 100 nM rapamycin are shown; these are the % reductions in growth/proliferation caused by the drug as compared to control treated cells. Data from Figure 1B are reproduced (filled bars) alongside independent repeat analyses (open bars). Data points represent means (+/- standard deviations) from five independent wells of cells. [file 1476-4598-10-19-S2.TIFF]
